# CXCR4-directed PET/CT with [^68^ Ga]Ga-pentixafor in solid tumors—a comprehensive analysis of imaging findings and comparison with histopathology

**DOI:** 10.1007/s00259-023-06547-z

**Published:** 2023-12-12

**Authors:** Niklas Dreher, Stefanie Hahner, Carmina T. Fuß, Wiebke Schlötelburg, Philipp E. Hartrampf, Sebastian E. Serfling, Andreas Schirbel, Samuel Samnick, Takahiro Higuchi, Alexander Weich, Constantin Lapa, Andreas Rosenwald, Andreas K. Buck, Stefan Kircher, Rudolf A. Werner

**Affiliations:** 1 Department of Nuclear Medicine, University Hospital of Würzburg, Oberdürrbacher Str. 6, 97080 Würzburg, Germany; 2 Department of Internal Medicine I, Endocrinology, University Hospital Würzburg, Würzburg, Germany; 3 Graduate School of Medicine, Dentistry and Pharmaceutical Sciences, Okayama University, Okayama, Japan; 4 Department of Internal Medicine II, Gastroenterology, University Hospital Würzburg, Würzburg, Germany; 5 Nuclear Medicine, Faculty of Medicine, University of Augsburg, Augsburg, Germany; 6 Institute of Pathology, University of Würzburg, Würzburg, Germany; 7 The Russell H. Morgan Department of Radiology and Radiological Science, Johns Hopkins University School of Medicine, Baltimore, MD USA; 8 Department of Diagnostic and Interventional Radiology and Nuclear Medicine, Division of Nuclear Medicine, Goethe University Frankfurt, University Hospital, Frankfurt, Germany

**Keywords:** [^68^ Ga]Ga-pentixafor, PET/CT, CXCR4, C-X-C motif chemokine receptor 4, Solid tumors, Theranostics, Radioligand therapy

## Abstract

**Background:**

C-X-C motif chemokine receptor 4 (CXCR4) is overexpressed in various solid cancers and can be targeted by CXCR4-directed molecular imaging. We aimed to characterize the in-vivo CXCR4 expression in patients affected with solid tumors, along with a comparison to ex-vivo findings.

**Methods:**

A total 142 patients with 23 different histologically proven solid tumors were imaged with CXCR4-directed PET/CT using [^68^ Ga]Ga-pentixafor (total number of scans, 152). A semi-quantitative analysis of the CXCR4-positive tumor burden including maximum standardized uptake values (SUV_max_) and target-to-background ratios (TBR) using blood pool was conducted. In addition, we performed histopathological staining to determine the immuno-reactive score (IRS) from patients’ tumor tissue and investigated possible correlations with SUV_max_ (by providing Spearman’s rho ρ). Based on imaging, we also assessed the eligibility for CXCR4-targeted radioligand therapy or non-radioactive CXCR4 inhibitory treatment (defined as more than five CXCR4-avid target lesions [TL] with SUV_max_ above 10).

**Results:**

One hundred three of 152 (67.8%) scans showed discernible uptake above blood pool (TBR > 1) in 462 lesions (52 primary tumors and 410 metastases). Median TBR was 4.4 (1.05–24.98), thereby indicating high image contrast. The highest SUV_max_ was observed in ovarian cancer, followed by small cell lung cancer, desmoplastic small round cell tumor, and adrenocortical carcinoma. When comparing radiotracer accumulation between primary tumors and metastases for the entire cohort, comparable SUV_max_ was recorded (*P* > 0.999), except for pulmonal findings (*P* = 0.013), indicative for uniform CXCR4 expression among TL. For higher IRS, a weak, but statistically significant correlation with increased SUV_max_ was observed (*ρ* = 0.328; *P* = 0.018). In 42/103 (40.8%) scans, more than five TL were recorded, with 12/42 (28.6%) exhibiting SUV_max_ above 10, suggesting eligibility for CXCR4-targeted treatment in this subcohort.

**Conclusions:**

In a whole-body tumor read-out, a substantial portion of prevalent solid tumors demonstrated increased and uniform [^68^ Ga]Ga-pentixafor uptake, along with high image contrast. We also observed a respective link between in- and ex-vivo CXCR4 expression, suggesting high specificity of the PET agent. Last, a fraction of patients with [^68^ Ga]Ga-pentixafor-positive tumor burden were rendered potentially suitable for CXCR4-directed therapy.

**Supplementary Information:**

The online version contains supplementary material available at 10.1007/s00259-023-06547-z.

## Introduction

C-X-C motif chemokine receptor 4 (CXCR4) is a transmembrane G-protein-coupled receptor crucially involved in tumor dissemination of varying malignancies [1, 2]. In this regard, analyses of tumor specimens have already demonstrated a substantial upregulation of this receptor subtype, which then triggered the use of the CXCR4-directed PET agent [^68^ Ga]Ga-pentixafor [3–5]. Those molecular imaging studies have revealed substantial uptake, including elevated maximum standardized uptake value (SUV_max_) or excellent image contrast (provided by increased target-to-background ratios [TBR]), particularly in hematological malignancies [6].

Beyond lymphoma or leukemia, increased chemokine receptor 4 expression on solid tumors was also linked to less favorable outcome, including ovarian, hepatocellular, neuroendocrine, or cholangiocarcinoma [7–9]. Previous studies on CXCR4-targeted PET/CT, however, included only a limited number of patients or scans. For instance, Vag et al. [10] reported on 21 subjects and revealed a discrepancy between the chemokine receptor profile observed in-vitro when compared to in-vivo findings provided by [^68^ Ga]Ga-pentixafor PET. A recent analysis enrolling 19 subjects with different solid cancers also showed that [^68^ Ga]Ga-pentixafor provided fluctuating uptake depending on the analyzed subtype [11]. Last, a recent study investigated a larger number of subjects, but only focused on a “hottest lesion” analysis (i.e., the target lesion [TL] with most intense uptake), while other PET-positive TL were not included [6].

In addition to investigations focusing on diagnosis, CXCR4-targeted PET/CT can be conducted to assess patient’s suitability for CXCR4-targeted therapies in a theranostic approach, e.g., radioligand therapy (RLT) or “cold” inhibitory drugs [12, 13]. CXCR4-directed RLT has already been applied to various hematological tumor entities [14–16]. Of note, a recent study demonstrated, for the first time, that this approach is also feasible in solid cancers by treating patients with desmoplastic small round cell tumor (DSRCT) [17]. Nevertheless, CXCR4-RLT may be limited to aggressive disease with virtually no other treatment options, given that CXCR4-directed “hot” treatment is associated with myeloablative effects due to CXCR4 expression on hematopoietic stem cells [18].

In the present investigation, we aimed to provide a comprehensive overview of intensity of uptake in 142 patients with 23 different kinds of solid cancers on 152 [^68^ Ga]Ga-pentixafor PET/CTs. For this purpose, we manually segmented the whole-body, chemokine receptor-avid tumor burden, which then allowed to sort tumor entities based on intensity of uptake. Regarding myeloablative effects of CXCR4-directed RLT, we additionally performed quantitative analyses of bone marrow (BM) receptor expression for the different entities. Furthermore, we assessed previous lines of therapy and concurrent diseases to examine their influence on tumor uptake and BM CXCR4-expression. We also conducted a correlation analysis between radiotracer accumulation and ex-vivo findings, including immunohistochemical staining of CXCR4-expression. Moreover, we aimed to determine patients eligible for a therapeutic approach based on the number of PET-positive TL and intensity of [^68^ Ga]Ga-pentixafor uptake in sites of disease.

## Material and methods

### Patient population

We retrospectively analyzed 152 [^68^ Ga]Ga-pentixafor-PET/CT scans of 142 patients with 23 different malignant solid tumors. Parts of this cohort have been described previously in [6, 11, 12, 17, 19–26], without assessing the CXCR4-avid tumor burden, PET-based eligibility for CXCR4-directed RLT, or correlation with histologically determined immunoreactive score (IRS) for such a large cohort. Table 1 provides an overview of diagnoses and further patient characteristics. Subjects signed written informed consent forms before examination. The local ethics committee waived the need for further approval due to the retrospective nature of this study (no. 20210726 02).

**Table 1:** Overview of investigated [^68^ Ga]Ga-PentixaFor PET/CTs and patient characteristics

Clinical variable		Patients (*n*)	PET/CT scans (*n*)	
			Positive	Negative
Tumor entity	ACC	34	30	5
	NEN	30	23	9
	SCLC	14	12	2
	DSRCT	10	14	2
	NSCLC	9	7	2
	HCC	8	4	4
	Pancreatic cancer	8	5	3
	Pleural mesothelioma	6	1	5
	Renal cell carcinoma	4	1	3
	Ovarian carcinoma	3	1	2
	CCC	3	2	1
	Prostate cancer	2	0	2
	Ewing sarcoma	1	1	0
	Osteosarcoma	1	1	0
	Mediastinal tumor*	1	1	0
	Colorectal carcinoma	1	0	2
	Leiomyosarcoma	1	0	1
	Thyroid cancer	1	0	1
	Paraganglioma	1	0	1
	Angiosarcoma	1	0	1
	Stromal sarcoma	1	0	1
	Neuroectodermal teratoma	1	0	1
	Liposarcoma	1	0	1
Age (in years)	59.5 (range, 8–89)			
Female		63/142 (44.4%)		
Prior therapies	None		30 (29.1%)	17 (34.7%)
	Surgery		53 (51.5%)	20 (40.8%)
	Radiation		21 (20.4%)	8 (16.3%)
	Systemic therapy		13 (12.6%)	5 (10.2%)
	Chemotherapy		64 (62.1%)	23 (46.9%)
	*1 line*		*21 (32.8%)*	*11 (47.8%)*
	*2 lines*		*15 (23.4%)*	*10 (43.5%)*
	≥ *3 lines*		*28 (43.8)*	*2 (8.7%)*
Concurrent illnesses	Cardiovascular		51 (49.5%)	28 (57.1%)
	Neurologic		9 (8.7%)	4 (8.2%)
	Secondary malignancy		11 (10.7%)	8 (16.3%)

ACC, adrenocortical carcinoma; CCC, cholangiocellular carcinoma; DSRCT, desmoplastic small round cell tumor; HCC, hepatocellular carcinoma; NEN, neuroendocrine neoplasia; NSCLC, non-small lung cell carcinoma; SCLC, small cell lung carcinoma

*Not otherwise specified

### Prior therapy lines and concurrent illnesses

In order to investigate the possible influence of previous therapy lines and concurrent illnesses on PET quantification, available clinical patient data was further examined. Previously performed therapy was collected including surgery, radiotherapy (RTx), and systemic tumor therapy, which we further subdivided into chemotherapy (CTx) and other systemic anti-tumor therapy. For CTx, we also differentiated patients according to the number of lines they had previously completed (1, 2, or ≥ 3). Regarding concurrent illnesses, we collected data on cardiovascular and neurologic diseases, as well as secondary malignancies.

### Imaging with [^68^ Ga]Ga-PentixaFor

Preparation of [^68^ Ga]Ga-pentixafor was conducted in house, as described before [5]. The whole-body PET/CTs (ranging from the vertex of the skull to the proximal thighs) were performed using a Siemens biograph mCT (64 or 128, Siemens Healthineers, Erlangen, Germany) after a median activity of 136.5 MBq (57–182) was administered 60 min beforehand. For attenuation correction and anatomic co-registration, low-dose CT scans were performed (120 keV, 512 × 512 matrix, 5 mm slices, increment: 30 mm/s, pitch index 0.8, and rotation time: 0.5 s). PET images were further corrected for random events and scatter.

### Image interpretation

Image analysis was performed by a single reader (ND) and verified by two experienced readers (SES, RAW). To further investigate CXCR4-positive tumor burden, the primary and TL in following body compartments were defined: bone, lymph node, liver, lung, and soft tissue. For each compartment, the three largest and/or most intense lesions were identified. Lesions were segmented using three-dimensional volumes of interest (VOI) with an isocontour threshold of 40%. We recorded the SUV_max_, the mean, and peak SUV (SUV_mean/peak_), as well as the volume (in mL) of every TL. As additional parameters, we examined the total measured tumor volume per scan (TV, in mL), for which we added up the measured volumes of the individual TL. Furthermore, we analyzed the fractional tumor activity (FTA), for which we multiplied SUV_mean_ and volume of each TL and then added up these values for each scan examined [27]. We also calculated target-to-background ratios (TBR) using blood pool as reference [6]. To assess bone marrow CXCR4 expression, we placed three-dimensional VOIs in the vertebral bodies C2, Th7, and L5 to obtain the respective SUV_mean_ and calculate the mean values for each patient [19].

### CXCR4 immunohistochemistry

Immunohistochemistry was conducted on 10% formalin fixed paraffin embedded tissue Sects. (3 µm) and subsequently scored as previously described [28]. An anti-CXCR4 rabbit polyclonal antibody (ab2074; Abcam, Cambridge, UK) was applied, followed by detection with the DAKO en vision system according to the manufacturer’s protocol.

Using light-microscopy, we quantitively analyzed the stained sections according to the IRS by Remmele and Stegner [29]. The proportion of CXCR4-positive cells was scored as follows: 0 (no positive cells), 1 (< 10% positive cells), 2 (10–50% positive cells), 3 (> 50–80% positive cells), and 4 (> 80% positive cells). In addition, the intensity of staining was graded: 0 (no color reaction), 1 (mild reaction), 2 (moderate reaction), and 3 (intense reaction). By multiplying the two scores, the respective IRS classification was obtained. To demonstrate a relationship between IRS and uptake of [^68^ Ga]Ga-pentixafor, we correlated these two parameters.

### Suitability for CXCR4-directed RLT

We performed a visual and quantitative analysis based on the [^68^ Ga]Ga-pentixafor-PET/CT to assess the hypothetical suitability for CXCR4-targeted RLT. Therefore, we defined the two criteria “intense tracer uptake” (average SUV_max_ of 10 in all segmented lesions) and “widespread disease” (presence of at least 5 CXCR4-positive TL) as respective prerequisites.

### Statistical analysis

All statistical analyses were performed using GraphPad Prism version 10.0.2 (GraphPad Software, San Diego, USA). Quantitative results are displayed as median and range (lowest–highest) or median and 5–95 percentile range. An outlier correction using the ROUT-method was conducted for BM uptake parameters. The non-parametric Mann–Whitney-test was performed to examine differences in tumor and BM uptake regarding prior therapies and concurrent illnesses. The non-parametric Kruskal–Wallis test, including a correction for multiple comparisons using the Dunn’s test, was performed to check for significant differences between tumor uptake in lesions of different organ compartments as well as for differences in tumor uptake for different numbers of prior chemotherapy lines and for differences in BM uptake between entities. To correlate immunohistochemical IRS and SUV parameters, the non-parametric Spearman’s rank correlation was conducted. A *p*-value of < 0.05 was considered to be statistically significant.

## Results

### [^68^ Ga]Ga-pentixafor obtains high image contrast in solid tumors

**Fig. 1:**
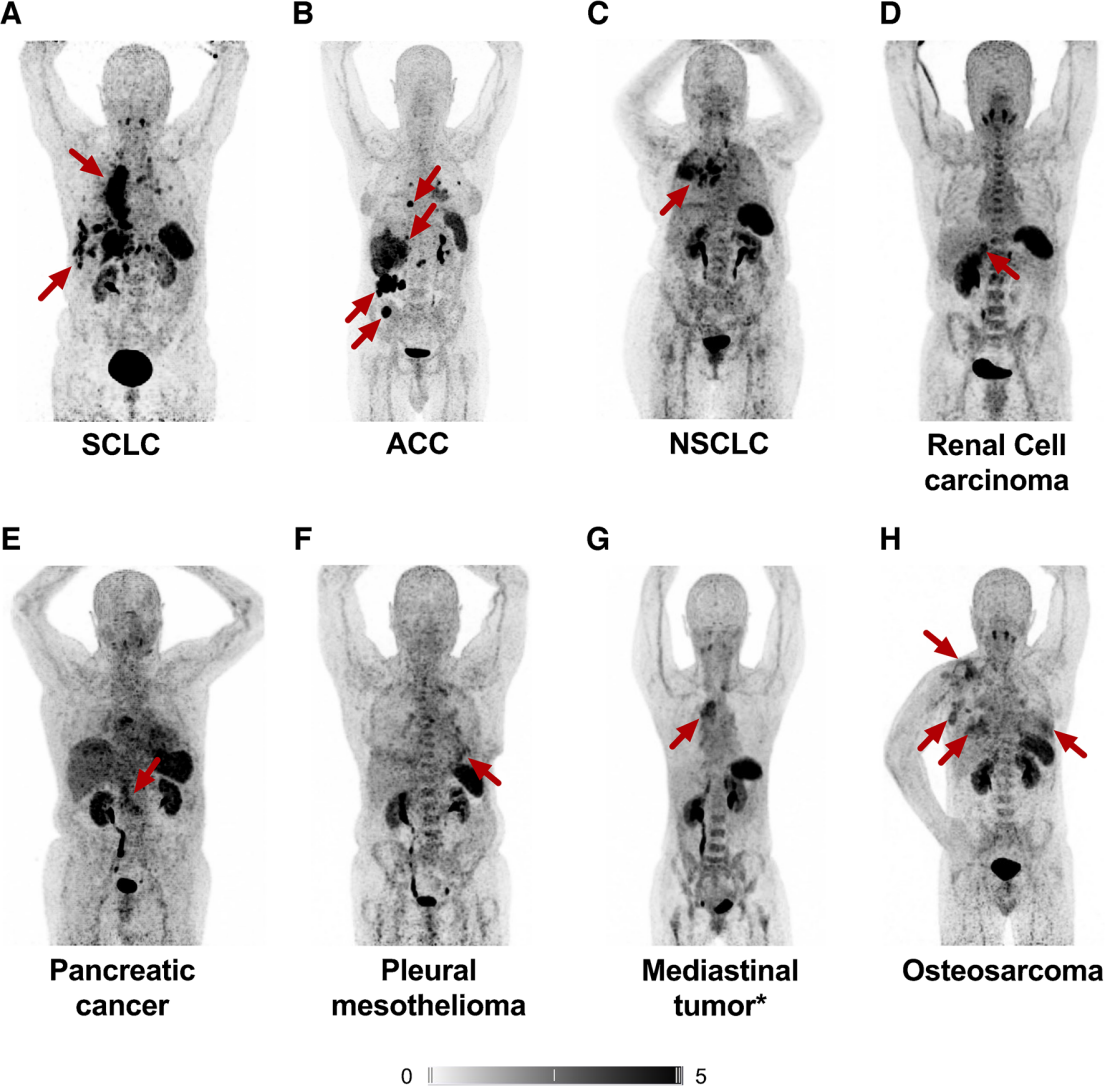
CXCR4-directed PET/CT using [^68^ Ga]Ga-PentixaFor in different solid tumor entities. Maximum intensity projections (MIP) are displayed. Red arrows highlight CXCR4-positive tumor lesions. ACC, adrenocortical carcinoma; NEN, neuroendocrine neoplasia; NSCLC, non-small cell lung carcinoma; SCLC, small cell lung carcinoma. *Not otherwise specified

**Fig. 2:**
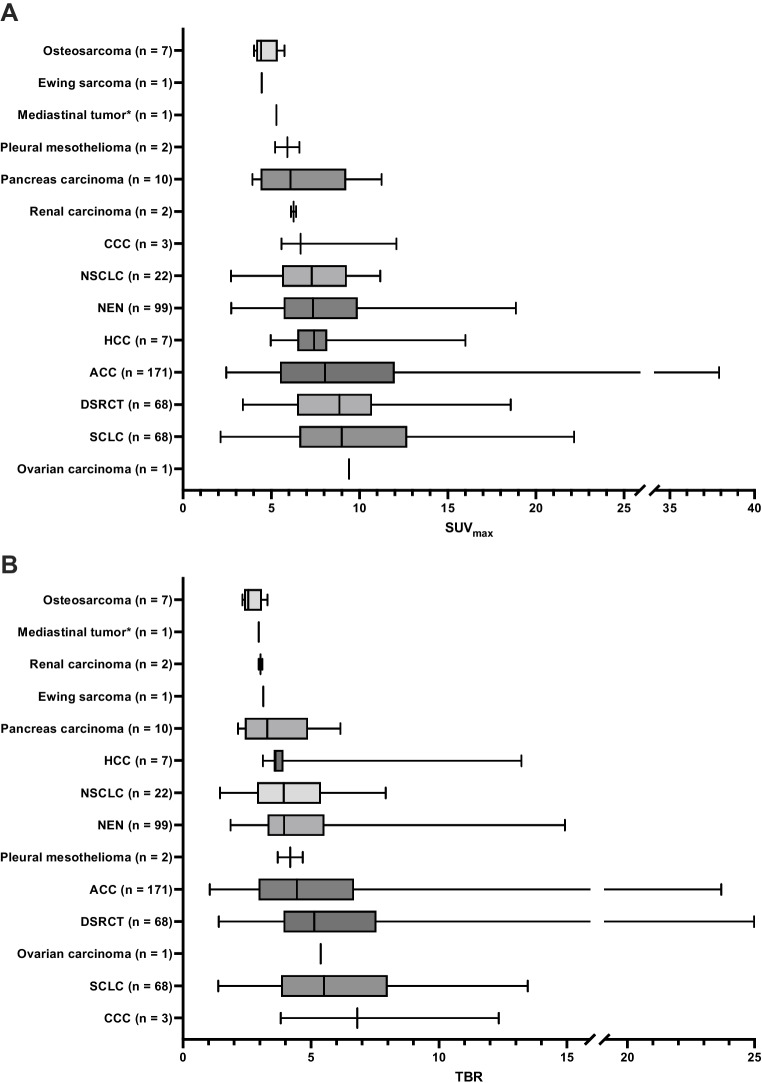
Results of the quantitative tumor burden analysis of [^68^ Ga]Ga-PentixaFor PET. Boxplots and whiskers display median and range of SUV_max_ (**A**) and TBR (**B**) for the examined entities. Quantities for all lesions per tumor entity are presented. ACC, adrenocortical carcinoma; CCC, cholangiocellular carcinoma; DSRCT, desmoplastic small round cell tumor; HCC, hepatocellular carcinoma; NEN, neuroendocrine neoplasia; NSCLC, non-small lung cell carcinoma; SCLC, small cell lung carcinoma. *Not otherwise specified

One hundred three of 152 (67.8%) scans demonstrated relevant uptake in the tumor burden and were therefore included in the further analyses. A total of 462 VOIs were placed around tumor lesions (Median 4 [1-13] per scan). Among these, 120/462 (26%) lesions were measured in the lymph nodes, 108/462 (23.4%) in the liver, and 96/462 (20.8%) in the soft tissue. Furthermore, 52/462 (11.3%) primary lesions were segmented, as well as 45/462 (9.7%) lung metastases and 41/462 (8.9%) bone lesions. Across all tumor entities, the VOIs yielded a median SUV_max_ of 7.89 (2.13–37.91), SUV_peak_ of 5.1 (1.5–28.13), and SUV_mean_ of 4.45 (1.33–24.52). SUV_mean_ of the blood pool was 1.79 (0.62–2.79), resulting in a median TBR of 4.4 (1.05–24.98). In addition, a median TV of 51.59 mL (1.18–935.1) and FTA of 263.5 (2.41–7910) were recorded. Based on SUV_max_, the highest uptake was observed in ovarian carcinoma, followed by small cell lung carcinoma (SCLC), desmoplastic small round cell carcinoma (DSRCT), and adrenal carcinoma (ACC). Figure 1 shows maximum intensity projections of [^68^ Ga]Ga-pentixafor PET/CTs, which serve as representative examples for the included tumor entities. Figure 2 displays median SUV_max_ and TBR based on all measured lesions for every examined tumor entity. Supplementary Table 1 displays the parameters subdivided in primary lesions and metastases as well as the quantities of these lesions per tumor entity. Assessing BM uptake in all patients of our cohort revealed a median SUV_mean_ of 1.73 (0.84–3.05). Comparing uptake between entities revealed no significant differences (*p* = 0.299). Supplementary Table 2 displays the median SUV_mean_ for each of the different tumor entities in our cohort.

### Uptake in metastatic lesions is comparable to primaries, indicative for uniform target expression among TL

Primary lesions showed a median SUV_max_ of 8.73 (4.85–26.92). Liver metastases had a SUV_max_ of 8.54 (3.58–27.40), bone metastases 8.11 (2.72–23.7), and soft tissue lesions of 7.8 (2.67–34.18). Lymph node metastases had a median SUV_max_ of 7.72 (3.09–37.91), followed by the least tracer-avid metastases in the lung (5.41 [2.13–22.9]).

**Fig. 3:**
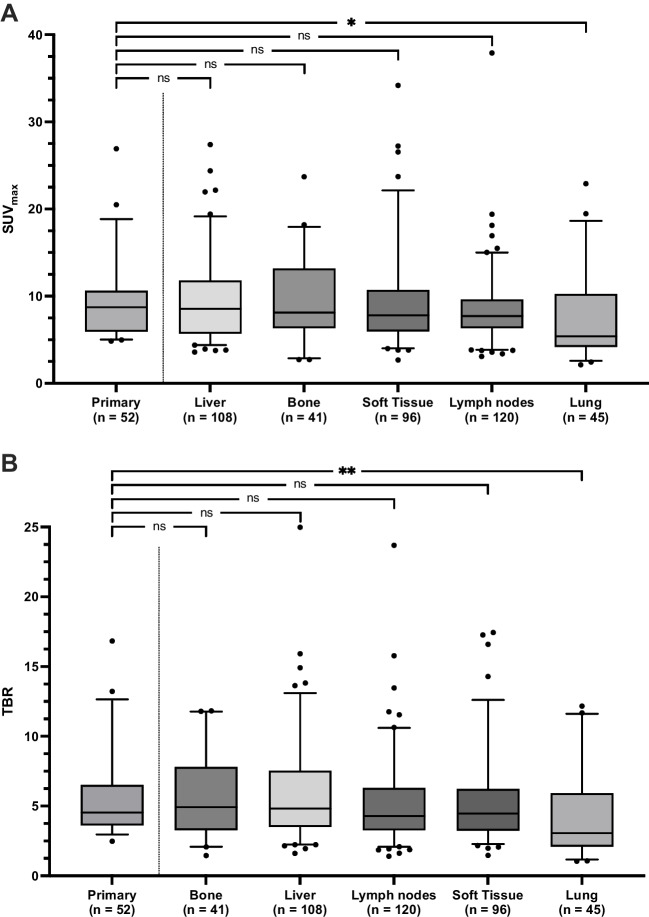
[.^68^ Ga]Ga-PentixaFor uptake in sites of disease across different compartments. **A** SUV_max_ and **B** TBR. Boxplots display median and interquartile range. Whiskers display 5–95 percentile range. * *P* < 0.05, ** *P* < 0.01

Comparing uptake in metastatic sites and primary lesions, SUV_max_ and TBR were not significantly different, except for lung TL (SUV_max_: *P* = 0.013; TBR: *P* = 0.007). Pulmonal uptake was also significantly lower in comparison to the other sites for TBR (*p* < 0.036) and SUV_max_ (*p* < 0.045; except for comparison with lymphonodal lesions, *P* = 0.053). Figure 3 displays comparisons between primary tumors and distant sites of disease (refer to Supplementary Table 3 for more detailed results).

### Patients pre-treated with CTx or RTx show no significant differences in CXCR4 expression, but lower BM uptake

Comparing SUV_max_ of tumor lesions in patients having received prior lines of CTx to patients without any previous lines of CTx, no significant difference could be observed (*p* = 0.478), which was also the case when comparing patients having received different numbers of CTx lines (1, 2, ≥ 3 lines, respectively; *p* = 0.287). Additionally, when comparing patients with or without prior RTx, we also observed no significant difference in SUV_max_ (*p* = 0.436). For concurrent illnesses, no significant difference in tumor uptake could be noted for cardiovascular diseases (*p* = 0.399), neurologic illnesses (*p* = 0.507), or secondary malignancies (*p* = 0.301), when compared to patients without respective illnesses. Assessing a potential influence of prior therapy lines on BM uptake, patients having received prior lines of CTx (*p* = 0.002) or RTx (*p* = 0.004) presented with a significantly lower SUV_mean_ in the BM. A significant difference in BM uptake between individuals with or without CXCR4-positive tumor burden could not be observed (*p* = 0.309).

### [^68^ Ga]Ga-pentixafor uptake correlates with ex-vivo CXCR4 expression, indicative for high specificity of the PET agent

**Fig. 4:**
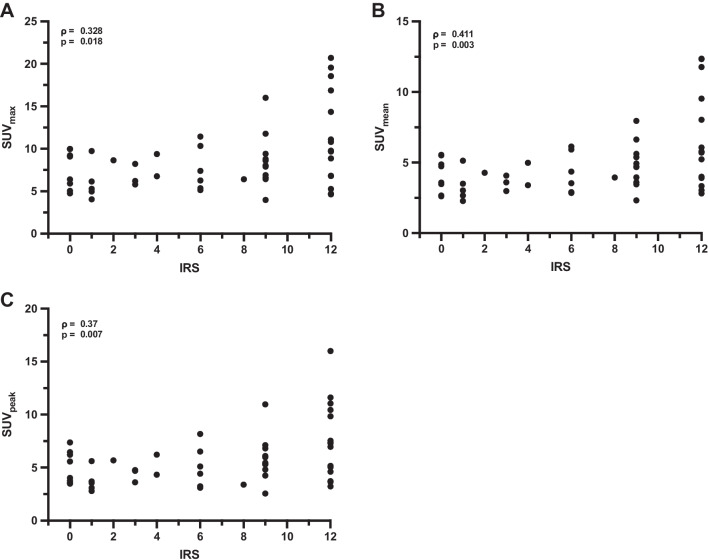
Correlation between immunoreactive score (IRS) and PET parameters. **A** SUV_max_, **B** SUV_mean_, and **C** SUV_peak_. Spearman’s rho and *p*-values are reported in the graphs

**Fig. 5:**
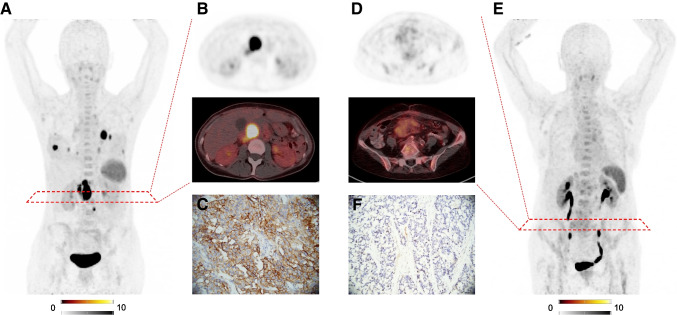
Concordance of CXCR4-directed immunohistochemistry (IHC) and [.^68^ Ga]Ga-pentixfor PET/CT. Patient with adrenal cortical carcinoma demonstrated increased uptake in sites of disease on maximum intensity projection (MIP, **A**) and transaxial PET/CT (**B**), along with high CXCR4 expression on IHC staining (**C**). SUV_max_ was 26.9 (TBR: 16.8) and respective immuno-reactive score (IRS) was 12. Patient in B was diagnosed with a neuroendocrine carcinoma, demonstrating no relevant uptake in the primary (**D**, **E** SUV_max_, 5.5; TBR: 2.5). IHC also revealed no relevant CXCR4 expression (IRS, 1). Magnification of IHC: × 400

One hundred three of 152 (67.8%) of scans demonstrated CXCR4-positive tumor burden, which were obtained from 95 different patients. For 52/95 (54.7%) patients, biopsy or surgical specimen was available. The respective entities were NEN (*n* = 17), ACC (*n* = 15), SCLC (*n* = 8), HCC, and pancreas carcinoma (*n* = 3, each) as well as renal cell carcinoma, ovarian cancer, pleural mesothelioma, osteosarcoma, CCC, and NSCLC (*n* = 1, each). Weak, but significant associations between increased IRS and SUV_max_ (*ρ* = 0.328; *P* = 0.018; Fig. 4A), SUV_mean_ (*ρ* = 0.411; *P* = 0.003; Fig. 4B), and SUV_peak_ (*ρ* = 0.37; *P* = 0.007; Fig. 4C) were recorded. Figure 5 shows respective cases of concordant findings of in- and ex-vivo CXCR4 expression in sites of disease.

### Up to 28% of patients are potentially eligible for CXCR4-directed treatment

A total of 42/103 (40.8%) of the scans had more than 5 different tumor lesions. Among those, 12/42 (28.6%) had a median SUV_max_ above 10 (ACC, 5/12 [41.7%]; neuroendocrine neoplasms, 4/12 [33.3%]; SCLC, 2/12 [16.7%]; DSCRT, 1/12 [8.3%]), thereby possibly rendering those patients eligible for CXCR4-targeted “cold” inhibitory drugs or RLT. Median TV was 151.1 mL (22.4–761.6) and median FTA 1008 (360–7910) for the considered patients.

## Discussion

Investigating the largest cohort of patients with solid cancers imaged with [^68^ Ga]Ga-pentixafor PET/CT to date, CXCR4-positive tumor burden was identified in more than two-thirds of the patients, along with high image contrast indicative for good read-out capabilities. Conducting a comprehensive tumor assessment, most intense uptake was observed in SCLC, DSRCT, and ACC, when investigating entities with a substantial number of examined lesions in our study. Assessing BM uptake in our cohort, no significant difference based on tumor subtypes could be identified, although those results may be potentially hampered for some of the entities due to the small number of subjects. When comparing chemokine receptor expression between primary lesions and metastases, no relevant differences in uptake could be determined (except for lung lesions). Furthermore, we observed no significant difference in tumor uptake when comparing patients with different therapies or concurrent diseases, whereas CXCR4 expression in the BM was significantly lower in patients having received prior CTx or RTx. Correlating multiple SUV parameters with immunohistochemically acquired IRS, a respective link between intensity of uptake and histopathologic findings was recorded, supporting the notion that [^68^ Ga]Ga-pentixafor PET achieves high specificity for the target. Last, more than 40% revealed a minimum of five target lesions positive on molecular imaging. Among such subjects with widespread disease, more than 28% also exhibited substantially high uptake levels, thereby potentially rendering those individuals eligible for “hot” or “cold” CXCR4 treatment.

Previous studies have already provided evidence that CXCR4 emerges as a suitable target for imaging and therapy in multiple solid tumors, including ACC, ovarian cancer, neuroendocrine tumors, or SCLC [9, 30–32]. Of note, those ex-vivo findings were further corroborated by our in-vivo imaging approach. First, our comprehensive tumor assessment yielded a substantially high median TBR > 4 when regarding TL from all tumor entities, indicative for an excellent image contrast when compared to physiological background activity. Second, we observed uniform and high receptor density on the primary and distant sites of disease in varying different cancer entities, except for lung lesions, although this exception could conceivably be explained by the limited spatial resolution of small nodules. Altogether, this finding could potentially indicate that CXCR4 RLT or “cold” chemokine receptor-mediated therapeutic approaches would most likely exert anti-tumor effects in all sites of disease throughout the body [12, 13]. Third, we also observed a correlation between different SUV parameters and IRS, thereby indicating that the PET signal provided by [^68^ Ga]Ga-pentixafor accurately reflects the chemokine receptor density on targeted tumor cells. In this regard, a previous analysis including a limited number of patients has also reported on an association between ex- and in-vivo findings after injection of this radiopharmaceutical [11]. In this study, we further elaborated on those preliminary findings by investigating more than 50 subjects with available histology. Of note, the observed association applied to both SUV_peak_ and SUV_max_, which are both independent from lesion size [33].

CXCR4-targeted RLT has already achieved durable response including partial or complete remission in patients with hematological malignancies [14, 15]. Our results, which revealed substantial tracer uptake in several entities and no significant difference in tumor uptake after different lines of therapy, support the notion that CXCR4-directed RLT could represent a viable option for extensively pre-treated patients in solid tumor entities. Nonetheless, such an approach would require stem cell backup because of its myeloablative effects, as CXCR4 is also part of the hematopoietic stem cell niche, thereby limiting this theranostic concept to patients that per se are in need of hematopoietic stem cell transplantation [34]. In patients with solid cancers, however, this would then be seen as major side effect, i.e., only suitable in a salvage setting in highly aggressive disease [34]. Additionally, the overall rate of patients suitable for RLT according to the applied criteria in our study was overall only 12/152 (8%). Despite these two rather unfavorable aspects regarding the practicability of CXCR4-RLT in solid tumors, one has to consider that a substantial portion of patients were diagnosed with difficult-to-treat tumor entities, including ACC. This tumor entity has a dismal prognosis [35] and even widely used therapeutic regimen has recently demonstrated heterogeneous outcome in selected cases, thereby emphasizing the urgent need for novel therapeutic approaches in those patients [36]. In this regard, a previous immunohistochemical-centered publication has already reported on substantial CXCR4 expression in half of the subjects, along with a relevant association of chemokine receptor upregulation with proliferation indices [37]. In line with those ex-vivo findings, another report investigating uptake on chemokine receptor PET in patients with ACC has also shown that up to 57% of the patients would have been eligible for CXCR4-RLT [21]. The present study indicates a lower number of patients suitable for treatment. As a possible explanation, the predefined criteria for (hypothetical) eligibility were rather strict in our study and less rigid predefinitions such as lower SUV_max_ may have led to a larger number of individuals suitable for CXCR4-targeted therapy. Furthermore, current clinical trials are investigating the appropriate amount of radiotherapeutic activity to achieve anti-tumor efficacy without exerting myeloablation (PTT101, EudraCT No. 2021–002364-43). Our finding of reduced BM tracer uptake in patients who had previously undergone CTx or RTx could also be of relevance in this regard. However, further investigation of the connection between pre-therapy uptake of the diagnostic PET tracer [^68^ Ga]Ga-pentixafor and myeloablative effects of CXCR4-directed RLT is needed. Nevertheless, once a desired activity range has been identified, the current study may provide a rationale to expand chemokine receptor theranostics beyond hematological malignancies toward solid tumors, which are currently not adequately covered by nuclear oncology. In this regard, based on our preliminary imaging findings, solid tumor entities including ACC, neuroendocrine neoplasms, and SCLC exhibited increased uptake with a relevant number of TL, potentially rendering those tumor entities eligible for RLT. Pleural mesothelioma, mediastinal tumor, Ewing-sarcoma, or osteosarcoma, however, may then rather not be well suited for treatment.

This study has limitations, including its retrospective design and limited number of patients for some of the examined tumor entities. Furthermore, we observed a high per-tumor variability in our study. Nonetheless, we herein report on the largest cohort of patients with solid tumors imaged with CXCR4-directed PET, along with substantial correlative indices between ex- and in-vivo findings. As such, the herein provided overview of subjects with solid cancers may therefore provide a roadmap to identify subjects eligible for CXCR4-directed imaging and therapy.

## Conclusion

In the largest cohort of subjects affected with solid cancers and imaged with [^68^ Ga]Ga-pentixafor PET to date, we observed elevated CXCR4 expression (provided by SUV_max_) and good image contrast (indicated by TBR) in two-thirds of the patients. In addition, ex-vivo chemokine receptor levels derived from IHC were linked to in-vivo uptake, suggesting that the PET signal indeed provides a read-out of the receptor density on the tumor cell surface. Of note, in-vivo chemokine receptor levels seem to be uniformly expressed, as no relevant uptake differences in the primary and metastases (with the exception of lung metastases) were observed, indicating that CXCR4-directed RLT would then exert anti-tumor effects in all lesions attributable to the underlying disease. As such, our preliminary findings may therefore provide a roadmap to identify subjects affected with solid tumors eligible for CXCR4-directed imaging and therapy.

### Supplementary Information

Below is the link to the electronic supplementary material.

Supplementary file1 (DOCX 30.9 KB)

## Data Availability

The datasets analyzed during the current study are available from the corresponding author on reasonable request.
