# Supplementary material for: CXCR4-directed PET/CT with [68 Ga]Ga-pentixafor in solid tumors—a comprehensive analysis of imaging findings and comparison with histopathology
Source: Eur J Nucl Med Mol Imaging. 2023 Dec 12;51(5):1383–94. doi: 10.1007/s00259-023-06547-z (PMC10957681; doi:10.1007/s00259-023-06547-z)
Supplement: Supplementary file 1 — Supplementary file1 (DOCX 30.9 KB) [file 259_2023_6547_MOESM1_ESM.docx]

**SUPPLEMENTARY**

**Supplementary Table 1.** **Results of the quantitative tumor burden analysis of [^68^Ga]Ga-PentixaFor PET.** Median and range are displayed for SUV_max_ and TBR for all segmented lesions each, as well as subdivided in primary manifestations and metastases. Entities are sorted by SUV_max_. ACC = Adrenocortical carcinoma; CCC = Cholangiocellular carcinoma; DSRCT = Desmoplastic small round cell tumor; HCC = Hepatocellular carcinoma; NEN = Neuroendocrine neoplasia; NSCLC = Non-small cell lung carcinoma; SCLC = Small cell lung carcinoma; *Not otherwise specified.

| Entity | Parameter | Compartment |  |
| --- | --- | --- | --- |
| All entities | SUV_max_ | All lesions (n = 462) | 7.89 (2.13 – 37.91) |
|  |  | Primary (n = 52) | 8.73 (4.85 – 26.92) |
|  |  | Metastases (n = 410) | 7.77 (2.13 – 37.91) |
|  | TBR | All lesions (n = 462) | 4.4 (1.05 – 24.98) |
|  |  | Primary (n = 52) | 4.54 (2.48 – 16.83) |
|  |  | Metastases (n = 52) | 4.4 (1.05 – 24.98) |
| Ovarian carcinoma | SUV_max_ | All lesions (n = 1) | 9.41 |
|  |  | Primary (n = 1) | 9.41 |
|  |  | Metastases (n = 0) | - |
|  | TBR | All lesions (n = 1) | 5.38 |
|  |  | Primary (n = 1) | 5.38 |
|  |  | Metastases (n = 0) | - |
| SCLC | SUV_max_ | All lesions (n = 68) | 8.99 (2.13 - 22.2) |
|  |  | Primary (n = 8) | 9.29 (5.35 - 17.2) |
|  |  | Metastases (n = 60) | 8.82 (2.13 - 22.2) |
|  | TBR | All lesions (n = 68) | 5.51 (1.38 - 13.5) |
|  |  | Primary (n = 8) | 4.97 (3 - 11) |
|  |  | Metastases (n = 60) | 5.6 (1.38 - 13.5) |
| DSRCT | SUV_max_ | All lesions (n = 68) | 8.86 (3.39 - 18.57) |
|  |  | Primary (n = 4) | 11.06 (6.3 - 12.94) |
|  |  | Metastases (n = 64) | 8.4 (3.39 - 18.57) |
|  | TBR | All lesions (n = 68) | 5.13 (1.4 - 24.98) |
|  |  | Primary (n = 4) | 7.79 (2.97 - 9.11) |
|  |  | Metastases (n = 64) | 5.04 (1.4 - 24.98) |
| ACC | SUV_max_ | All lesions (n = 171) | 8.05 (2.45 - 37.91) |
|  |  | Primary (n = 8) | 12.1 (7.1 - 26.92) |
|  |  | Metastases (n = 163) | 7.94 (2.45 - 37.91) |
|  | TBR | All lesions (n = 171) | 4.45 (1.05 - 23.69) |
|  |  | Primary (n = 8) | 6.38 (2.96 - 16.83) |
|  |  | Metastases (n = 163) | 4.42 (1.05 - 23.69) |
| HCC | SUV_max_ | All lesions (n = 7) | 7.43 (4.97 - 16) |
|  |  | Primary (n = 4) | 6.8 (4.97 - 16) |
|  |  | Metastases (n = 3) | 7.52 (7.43 - 8.16) |
|  | TBR | All lesions (n = 7) | 3.62 (3.12 - 13.2) |
|  |  | Primary (n = 4) | 3.64 (3.12 - 13.2) |
|  |  | Metastases (n = 3) | 3.62 (3.57 - 3.92) |
| NEN | SUV_max_ | All lesions (n = 99) | 7.37 (2.73 – 18.9) |
|  |  | Primary (n = 12) | 7.1 (5.05 – 10.6) |
|  |  | Metastases (n = 87) | 7.37 (2.73 – 18.9) |
|  | TBR | All lesions (n = 99) | 3.95 (1.86 – 14.9) |
|  |  | Primary (n = 12) | 3.76 (2.48 – 7.74) |
|  |  | Metastases (n = 87) | 4.04 (1.86 – 14.9) |
| NSCLC | SUV_max_ | All lesions (n = 22) | 7.3 (2.72 - 11.2) |
|  |  | Primary (n = 7) | 8.74 (4.85 - 10.3) |
|  |  | Metastases (n = 15) | 7.14 (2.72 - 11.2) |
|  | TBR | All lesions (n = 22) | 3.94 (1.45 - 7.92) |
|  |  | Primary (n = 7) | 4.25 (3.07 - 7.92) |
|  |  | Metastases (n = 15) | 3.22 (1.45 - 6.78) |
| CCC | SUV_max_ | All lesions (n = 3) | 6.67 (5.58 - 12.1) |
|  |  | Primary (n = 2) | 8.84 (5.58 - 12.1) |
|  |  | Metastases (n = 1) | 6.67 |
|  | TBR | All lesions (n = 3) | 6.81 (3.82 - 12.3) |
|  |  | Primary (n = 2) | 8.08 (3.82 - 12.3) |
|  |  | Metastases (n = 1) | 6.81 |
| Renal cell carcinoma | SUV_max_ | All lesions (n = 2) | 6.27 (6.12 - 6.41) |
|  |  | Primary (n = 0) | - |
|  |  | Metastases (n = 2) | 6.27 (6.12 - 6.41) |
|  | TBR | All lesions (n = 2) | 3.03 (2.96 - 3.1) |
|  |  | Primary (n = 0) | - |
|  |  | Metastases (n = 2) | 3.03 (2.96 - 3.1) |
| Pancreatic cancer | SUV_max_ | All lesions (n = 10) | 6.1 (3.93 – 11.3) |
|  |  | Primary (n = 4) | 8.72 (5.8 – 11.3) |
|  |  | Metastases (n = 6) | 4.96 (3.93 – 9.32) |
|  | TBR | All lesions (n = 10) | 3.3 (2.15 – 6.15) |
|  |  | Primary (n = 4) | 4.36 (3.1 – 6.15) |
|  |  | Metastases (n = 6) | 2.71 (2.15 – 5.09) |
| Pleural mesothelioma | SUV_max_ | All lesions (n = 2) | 5.91 (5.22 - 6.6) |
|  |  | Primary (n = 1) | 5.22 |
|  |  | Metastases (n = 1) | 6.6 |
|  | TBR | All lesions (n = 2) | 4.19 (3.7 - 4.68) |
|  |  | Primary (n = 1) | 3.7 |
|  |  | Metastases (n = 1) | 4.68 |
| Mediastinal tumor* | SUV_max_ | All lesions (n = 1) | 5.29 |
|  |  | Primary (n = 1) | 5.29 |
|  |  | Metastases (n = 0) | - |
|  | TBR | All lesions (n = 1) | 2.96 |
|  |  | Primary (n = 1) | 2.96 |
|  |  | Metastases (n = 0) | - |
| Ewing sarcoma | SUV_max_ | All lesions (n = 1) | 4.46 |
|  |  | Primary (n = 0) | - |
|  |  | Metastases (n = 1) | 4.46 |
|  | TBR | All lesions (n = 1) | 3.14 |
|  |  | Primary (n = 0) | - |
|  |  | Metastases (n = 1) | 3.14 |
| Osteosarcoma | SUV_max_ | All lesions (n = 7) | 4.43 (4.03 - 5.75) |
|  |  | Primary (n = 0) | - |
|  |  | Metastases (n = 7) | 4.43 (4.03 - 5.75) |
|  | TBR | All lesions (n = 7) | 2.55 (2.32 - 3.3) |
|  |  | Primary (n = 0) | - |
|  |  | Metastases (n = 7) | 2.55 (2.32 - 3.3) |

**Supplementary Table 2. Bone marrow uptake in the different examined tumor entities.** Median SUV_mean_ and range (only shown for entities with >1 examined lesion) are displayed. Entities are sorted by SUV_mean_. ACC = Adrenocortical carcinoma; CCC = Cholangiocellular carcinoma; DSRCT = Desmoplastic small round cell tumor; HCC = Hepatocellular carcinoma; NEN = Neuroendocrine Neoplasia; NSCLC = Non small lung cell carcinoma; SCLC = Small cell lung carcinoma; *Not otherwise specified.

| Tumor entity | SUV_mean_ Bone marrow |
| --- | --- |
| Ewing sarcoma | 2.42 |
| Leiomyosarcoma | 2.16 |
| Angiosarcoma | 2.08 |
| NSCLC | 1.94 (1.35 – 3.05) |
| Osteosarcoma | 1.9 |
| Pleural mesothelioma | 1.885 (1.63 – 2.64) |
| Colorectal carcinoma | 1.885 (1.84 – 1.93) |
| Liposarcoma | 1.87 |
| Paraganglioma | 1.82 |
| ACC | 1.785 (0.91 – 2.76) |
| HCC | 1.76 (1.55 – 2.23) |
| NEN | 1.76 (0.9 – 2.6) |
| Ovarian cancer | 1.75 (1.36 – 2.17) |
| CCC | 1.74 (1.71 – 1.86) |
| Thyroid cancer | 1.72 |
| Mediastinal tumor* | 1.61 |
| Prostate cancer | 1.565 (1.19 – 1.94) |
| Pancreas carcinoma | 1.48 (1.17 – 2.61) |
| Renal cell carcinoma | 1.46 (1.19 – 2.65) |
| DSRCT | 1.42 (0.85 – 2.91) |
| SCLC | 1.385 (0.84 – 2.17) |
| Neuroectodermal teratoma | 1.31 |
| Stromal sarcoma | 1.18 |

**Supplementary Table 3. Uptake sorted by primary lesions and different metastatic sites derived from [^68^Ga]Ga-PentixaFor PET.** For each compartment, SUV_max_ and TBR are reported. Significant parameters are marked in bold. LN = lymph nodes; ST = soft tissue.

| Localization | | Parameter | Median (range) | P-values | | | | | |
| --- | --- | --- | --- | --- | --- | --- | --- | --- | --- |
|  |  |  |  | *Metastases* | | | | | Primary |
|  |  |  |  | Lung | LN | ST | Bone | Liver |  |
| Primary | | SUV_max_ | 8.73 (4.85 – 26.92) | **0.013** | >0.999 | >0.999 | >0.999 | >0.999 | - |
|  |  | TBR | 4.56 (2.48 – 16.83) | **0.007** | >0.999 | >0.999 | >0.999 | >0.999 | - |
| *Metastases* | Liver | SUV_max_ | 8.54 (3.58 – 27.40) | **0.003** | >0.999 | >0.999 | >0.999 | - | >0.999 |
|  |  | TBR | 4.82 (1.61 – 24.98) | **0.0003** | >0.999 | >0.999 | >0.999 | - | >0.999 |
|  | Bone | SUV_max_ | 8.11 (2.72 – 23.7) | **0.018** | >0.999 | >0.999 | - | >0.999 | >0.999 |
|  |  | TBR | 4.92 (1.45 – 11.82) | **0.003** | >0.999 | >0.999 | - | >0.999 | >0.999 |
|  | ST | SUV_max_ | 7.8 (2.67 – 34.18) | **0.045** | >0.999 | - | >0.999 | >0.999 | >0.999 |
|  |  | TBR | 4.46 (1.46 – 17.44) | **0.014** | >0.999 | - | >0.999 | >0.999 | >0.999 |
|  | LN | SUV_max_ | 7.72 (3.09 – 37.91) | 0.053 | - | >0.999 | >0.999 | >0.999 | >0.999 |
|  |  | TBR | 4.28 (1.4 – 23.69) | **0.036** | - | >0.999 | >0.999 | >0.999 | >0.999 |
|  | Lung | SUV_max_ | 5.41 (2.13 – 22.9) | - | 0.053 | **0.045** | **0.018** | **0.003** | **0.018** |
|  |  | TBR | 3.06 (1.05 – 12.16) | - | **0.036** | **0.014** | **0.003** | **0.0003** | **0.007** |
